# The role of psychiatric nurses in nutritional care for treatment-resistant depression: a relapse-prevention perspective

**DOI:** 10.3389/fnut.2026.1961751

**Published:** 2026-09-03

**Authors:** Agnieszka Mechlińska, Natalia Sudoł, Dorota Pazda-Juda, Wiesław Jerzy Cubała

**Affiliations:** 1 Department of Psychiatry, Faculty of Medicine, Medical University of Gdańsk, Gdańsk, Poland; 2 Department of Psychiatry, UCC Gdańsk, Gdańsk, Poland

**Keywords:** dietary intervention, nutritional care, psychiatric nursing, relapse prevention, treatment-resistant depression

## Abstract

Treatment-resistant depression (TRD) is associated with persistent symptoms, impaired functioning, physical comorbidity and a high risk of recurrence, making continuation and maintenance care particularly important. Nutrition may influence several modifiable aspects of recovery, including cardiometabolic health, daily functioning, self-care and treatment adherence, although evidence for a direct effect on depressive recurrence remains limited. This review examines the role of nutritional care within TRD relapse prevention, with particular attention to psychiatric nursing practise. Available dietary intervention studies in depression suggest potential benefits for depressive symptoms and broader health outcomes, but most have focused on acute treatment rather than recurrence or maintenance, and data specific to TRD remain sparse. Evidence for nurse-delivered nutritional interventions is also limited, although studies in broader psychiatric populations indicate that dietary assessment, education, behavioural support and follow-up can be incorporated into mental health care. In TRD, psychiatric nurses may contribute through nutritional risk screening, monitoring of appetite, intake, weight and physical health, reinforcement of agreed dietary recommendations, and coordination of specialist referral. Clear professional boundaries are required when malnutrition, eating disorders, refeeding risk, therapeutic diets or clinically significant nutrient–drug interactions are present. Nutritional care should therefore be regarded as an adjunct to established TRD treatment and integrated into multidisciplinary continuation and relapse-prevention care rather than used as a stand-alone intervention.

## 1 Introduction

TRD is commonly defined as an inadequate response to at least two antidepressant treatments given at an adequate dose and for an adequate duration, although contemporary consensus increasingly views treatment resistance as a multidimensional and longitudinal clinical problem rather than a discrete biological subtype (1–4). This distinction is relevant to relapse prevention. Patients who require multiple treatment steps may enter remission with persistent residual symptoms, functional impairment, medical comorbidity, treatment fatigue and accumulated psychosocial burden. Continuation and maintenance care therefore extends beyond prevention of a threshold-defined depressive episode and includes consolidation of recovery, reduction of residual symptoms, preservation of function, early detection of deterioration and management of modifiable factors that repeatedly destabilise the patient (5–7). Long-term management is especially relevant for patients with TRD or difficult-to-treat depression who have achieved full or partial remission, are transitioning from acute treatment to continuation care, or are sufficiently stable to engage in self-management.

Nutrition is one such factor. Depression may affect appetite, eating behaviour, meal planning, reward processing and concentration, all of which can interfere with adequate nutrition and everyday self-care. Psychotropic medicines may alter appetite, body weight, glucose regulation, gastrointestinal function or taste. Food insecurity may increase reliance on inexpensive energy-dense foods, while social isolation may disrupt normal meal routines. At the other extreme, restrictive self-directed diets, prolonged fasting, multiple supplement use and rapid weight-loss attempts may contribute to nutritional inadequacy, binge–restriction cycles or clinically relevant interactions. These problems may complicate treatment through poor adherence, adverse effects, physical illness, fatigue or sleep disturbance (3, 4, 8–11). Most dietary intervention studies in depression have, however, focused on acute symptom improvement rather than recurrence or maintenance, and few have specifically recruited well-characterised TRD populations. Nutritional recommendations in this setting should therefore reflect both the available evidence and the patient’s clinical condition, safety and ability to implement change.

Psychiatric nurses have regular contact with patients across different stages of treatment and can contribute to assessment of self-care, daily functioning, medication and supplement use, physical health, emerging deterioration and referral needs. Nutritional nursing care does not replace dietetic assessment or medical treatment. Its main contribution lies in early recognition, practical support, monitoring and continuity of care. These functions are relevant in outpatient mood-disorder services, community mental-health care, post-discharge follow-up and specialist interventional psychiatry. By contrast, severe depressive deterioration with inability to maintain oral intake, medical instability, suspected refeeding risk, psychosis, catatonia or imminent suicide risk requires urgent specialist management rather than routine nutritional or lifestyle counselling. This paper translates current nutritional psychiatry evidence into practical guidance for psychiatric nursing in TRD continuation and relapse-prevention care, while distinguishing routine food-based support from clinician-directed supplementation and specialist dietary treatment (9–13).

## 2 Literature search and selection

The literature was last updated in August 2026. PubMed and Google Scholar were used to identify publications relevant to nutritional care in treatment-resistant depression and the role of psychiatric nurses. The review included literature on TRD and relapse prevention, dietary and nutritional interventions in depression, psychiatric nursing and nurse-led lifestyle interventions, as well as nutritional screening, malnutrition, refeeding syndrome, eating-disorder screening, and nutraceuticals. Priority was given to clinical guidelines, consensus statements, randomised controlled trials, systematic reviews, and meta-analyses. As evidence specifically addressing nurse-delivered nutritional care in TRD is limited, studies involving broader populations with depression or serious mental illness were also considered when relevant to the clinical issues discussed in this review. Reference lists of key publications were examined to identify additional relevant studies. Given the narrative nature of the review, no formal systematic-review protocol or risk-of-bias assessment was applied.

## 3 Why nutrition belongs in a TRD relapse-prevention strategy

Relapse after TRD is influenced by residual symptoms, illness history, psychosocial stress, sleep disturbance, medical comorbidity, substance use, treatment discontinuation and disruption of daily routines (3, 5, 6). Nutritional care may address several modifiable factors within this broader recovery context. Regular meals can provide structure to the day, adequate nutrient intake may prevent or correct nutrition-related contributors to fatigue, and diets based largely on minimally processed, fibre-rich foods may support cardiometabolic health. Collaborative meal planning may also support self-efficacy and daily functioning. These outcomes may be clinically relevant even if an effect on depressive recurrence has not been established.

Evidence from acute-treatment studies is encouraging but remains limited. The SMILES, HELFIMED and AMMEND trials reported greater improvement in depressive symptoms with structured dietary interventions, generally based on Mediterranean-style principles, than with comparator conditions (14–16). A systematic review, however, identified only five whole-diet intervention studies in patients with diagnosed depression, with substantial heterogeneity and small sample sizes limiting generalisability (17). Prospective observational studies have also associated higher diet quality and lower dietary inflammatory potential with more favourable subsequent depression outcomes, although causality cannot be inferred and residual social, behavioural and health-related confounding remains likely (18, 19).

The PREDIDEP trial provides the most direct evidence relevant to recurrence prevention. In adults recovered from depression, a two-year Mediterranean diet intervention enriched with extra-virgin olive oil did not significantly reduce depressive recurrence compared with usual care. Improvements in subsyndromal depressive symptoms were nevertheless observed at several time points, and remote dietary support appeared to facilitate adherence (20, 21). Improvements were also reported in selected quality-of-life domains (22). These findings suggest that nutritional intervention may support recovery and residual symptom management without constituting an established treatment for prevention of recurrence. It should complement continuation pharmacotherapy, relapse-focused psychotherapy and other established approaches rather than replace them (5, 6).

A further rationale is the close relationship between depression and physical illness. Obesity, diabetes, dyslipidaemia, cardiovascular disease, gastrointestinal disorders and chronic inflammatory conditions are common in difficult-to-treat populations and may adversely affect quality of life, treatment tolerability and long-term health. Nutritionally adequate eating patterns may therefore provide benefit independently of any direct antidepressant effect through improved cardiometabolic health and self-management. In patients whose individual pattern of deterioration includes disrupted sleep, loss of energy, irregular eating or poorer management of physical illness, changes in nutritional routines may also provide an early signal of declining function.

## 4 Integrating nutritional care into mental health nursing practise

Mental health nursing includes therapeutic engagement, care coordination and support for recovery and self-management in addition to symptom monitoring and medication-related care. A therapeutic relationship can facilitate shared decision-making and engagement with longer-term treatment. This is relevant to nutrition and other health behaviours, where individual barriers need to be identified and behavioural changes often require repeated support rather than one-off advice (23, 24).

Mental health nurses have also participated in structured interventions targeting physical health and lifestyle behaviours. Randomised trials have included nurse-led programmes combining healthy-eating education with exercise and motivational support, as well as lifestyle self-management interventions involving repeated follow-up. Evidence for their effectiveness remains inconsistent, particularly for weight-related outcomes (25). Usher et al. demonstrated that nutritional education could be incorporated into nurse-led lifestyle support for people with serious mental illness alongside exercise and motivational strategies, although the 12-week intervention did not significantly improve weight-related outcomes compared with usual care (26). In a larger 12-month trial, Holt et al. combined dietary and physical-activity education with behavioural support, individual goal setting and regular follow-up. Mental health nurses contributed substantially to delivery, although the programme was not exclusively nurse-led. No significant improvements were observed in weight, dietary intake, physical activity or metabolic outcomes (27). These studies support the feasibility of incorporating nutritional and lifestyle support into mental-health care but provide little evidence that education alone produces sustained clinical change.

More recent studies have examined broader nutritional roles for psychiatric mental health nurses (PMHNs), including assessment of eating behaviours and cardiometabolic parameters, motivational interviewing, repeated follow-up and family involvement. Such interventions suggest a role that extends beyond information provision to identifying nutritional problems and supporting implementation of agreed behavioural changes. PMHN-delivered dietary counselling has also combined structured education with regular reinforcement; although it was not superior to conventional nutritional counselling, favourable changes in some dietary behaviours were reported (28). Overall, the available evidence supports nutritional care as a feasible component of psychiatric nursing, but its likely contribution lies in ongoing assessment, behavioural support and continuity rather than dietary education in isolation.

These functions form the basis of the practical nursing responses described in Table 1 for nutritional problems that may arise during TRD continuation and maintenance care.

**Table 1:** Nutritional assessment and nursing response in patients with treatment-resistant depression.

Clinical area/problem	Nursing assessment	Nursing role and initial response	When further assessment is needed
Reduced appetite or inadequate intake	Appetite change, duration, meal frequency, hydration, recent weight change, GI symptoms, medication changes	Identify barriers to adequate intake; support regular eating opportunities and simple, acceptable food options; monitor intake and weight trajectory	Inability to maintain intake, dehydration, substantial unintentional weight loss, malnutrition or refeeding risk.
Nutritional adequacy and refeeding risk	Recent markedly reduced or negligible intake; prolonged fasting; recent substantial weight loss; low BMI; signs of muscle or subcutaneous fat loss; available phosphate, potassium and magnesium results; conditions associated with increased refeeding risk.	Recognise and document refeeding-risk factors, communicate concerns to the multidisciplinary team, avoid encouraging rapid increases in energy intake before assessment, and support monitoring of intake, weight, fluid balance, vital signs and relevant laboratory results according to the agreed care plan.	Dietetic/medical assessment for significant malnutrition, prolonged minimal intake, substantial recent weight loss, electrolyte abnormalities or other features suggesting refeeding risk
Irregular eating pattern	Skipped meals, prolonged fasting, nocturnal eating, changes from usual routine, relationship with mood and daily functioning	Support restoration of a feasible meal structure; identify low-effort alternatives for periods of reduced energy or executive functioning	Progressive restriction, recurrent binge-restriction pattern, or eating changes accompanied by psychiatric deterioration
Increased appetite or weight gain	Appetite changes, medication effects, eating pattern, binge-eating symptoms, sleep and relevant metabolic indicators	Explore possible contributors without weight stigma; reinforce general health behaviours and monitor trajectory rather than weight alone	Rapid or unexplained weight change, metabolic deterioration, suspected binge-eating disorder
Eating-disorder symptoms or restrictive practises	Restriction, bingeing, purging, rigid food rules, body-image concerns, previous ED, intentional fasting or restrictive diets	Use non-judgemental communication; avoid reinforcing restriction; prioritise nutritional adequacy and facilitate further assessment	Purging, syncope, rapid weight loss, electrolyte abnormalities, marked restriction or other clinically significant ED symptoms
Cardiometabolic risk	Weight trajectory, blood pressure and clinically indicated glucose/HbA1c and lipid results; relevant comorbidities	Reinforce general cardiometabolic dietary principles and support agreed health goals; coordinate routine monitoring	Significant deterioration, recurrent hypo−/hyperglycaemia or need for medical/dietetic treatment
GI symptoms affecting intake	Nausea, vomiting, constipation, diarrhoea, dysphagia, pain and possible relationship with medication	Identify impact on eating and hydration; document symptoms and reinforce appropriate general measures	Persistent vomiting, dysphagia, GI bleeding, severe pain or dehydration
Frailty or complex medical comorbidity	Age-related functional limitations, low appetite, swallowing or dental problems, relevant renal, hepatic or other medical disease	Adapt general support to functional capacity and existing treatment; prioritise nutritional adequacy and avoid applying generic dietary advice when condition-specific restrictions may be relevant	Dysphagia, progressive frailty, significant loss of intake or weight, or need for condition-specific medical nutrition therapy
Food insecurity or functional barriers	Food affordability and availability, storage, transport, social support and ability to organise meals	Adapt goals to available resources; simplify recommendations; coordinate practical or social support	Recurrent lack of access to adequate food, major functional impairment or safeguarding concerns
Supplement and nutraceutical use	Product, dose, indication, source, duration, concurrent medicines and prescriber awareness	Reconcile and document supplement use; reinforce that supplements are not substitutes for adequate nutrition or established treatment	Potential interaction, adverse effects, unknown composition, high-dose/complex regimens or suspected toxicity
Changes potentially associated with psychiatric deterioration	New changes in appetite, meal regularity, intake and self-care together with mood, anhedonia, sleep and functioning	Treat nutritional change as a potential clinical signal; explore context, support adequate intake and communicate relevant changes	Worsening depression, suicidality, marked loss of self-care or inability to maintain intake

The recommendations presented in this table represent author-synthesised clinical guidance based on evidence from related areas, including psychiatric nursing practise, nutritional and malnutrition screening, refeeding-risk management, and eating-disorder assessment. Given the limited TRD-specific evidence, these recommendations should be interpreted as practical clinical guidance rather than interventions directly validated in TRD populations.

## 5 Practical nutritional assessment and nursing recommendations

### 5.1 Clinical context and treatment phase

Nutritional priorities depend on the patient’s current clinical phase. During severe depressive deterioration, preservation of adequate food and fluid intake and prevention of further physical decline may take precedence over longer-term dietary change. In partial remission, attention may shift towards restoring regular eating and addressing persistent appetite or functional difficulties. During stable continuation or maintenance care, dietary quality, self-management and cardiometabolic health can be considered more broadly. Recent changes in psychotropic or other relevant medicines should also be recorded because they may affect appetite, weight, gastrointestinal symptoms or metabolic parameters.

### 5.2 Appetite, intake and meal regularity

Nutritional assessment should begin with a brief, concrete and non-judgemental history of current food and fluid intake. Relevant questions include what the patient usually eats and drinks, how often they eat, whether appetite or food preferences have changed, whether meals are frequently missed, and whether prolonged fasting, nocturnal eating or loss-of-control eating occurs. Symptoms that may interfere with intake should also be explored, including nausea, constipation, dysphagia, early satiety, gastrointestinal discomfort, dental problems and altered taste. Meal regularity should be interpreted in relation to the patient’s usual routine, treatment and level of functioning. The purpose is not to impose a fixed meal schedule but to identify patterns that compromise adequate intake or indicate deterioration in self-care. Increasing meal omission, progressive appetite loss or reduced ability to organise food may accompany worsening depression or functional decline and should be considered alongside other warning signs of relapse. Where available, validated malnutrition screening tools may support risk identification but should not replace clinical assessment (29). Markedly reduced intake or prolonged periods of minimal intake should also raise concern about refeeding risk, especially when accompanied by low BMI, substantial recent weight loss, loss of muscle or subcutaneous fat, or abnormalities in serum phosphate, potassium or magnesium. Particular caution is warranted in patients with eating disorders, severe mental illness associated with prolonged inadequate intake, alcohol or substance-use disorders, or other causes of prolonged undernutrition. Normal prefeeding electrolyte concentrations do not exclude depleted total-body stores. Suspected refeeding risk warrants medical and dietetic assessment before a substantial increase in energy intake is undertaken (30).

### 5.3 Anthropometry and weight trajectory

Body weight and height should form part of the basic nutritional assessment and allow calculation of body mass index (BMI). Current weight should be interpreted alongside recent trajectory, including the magnitude and rate of weight loss or gain, because BMI alone may fail to identify nutritional deterioration. In patients who are underweight, malnourished or suspected of having a restrictive eating disorder, measurements should be obtained consistently. Where possible, weight should be measured on the same calibrated scale, without shoes and in underwear or light clothing, after voiding and at a similar time of day. Repeated measurements should be obtained under comparable conditions to reduce variation unrelated to true changes in body weight. Weight, BMI and weight trajectory should be interpreted together with current intake, physical symptoms and medical stability. Rapid or progressive weight loss, very low body weight or continued deterioration requires further clinical assessment, including consideration of malnutrition, refeeding risk and the need for specialist nutritional or eating-disorder care (30).

### 5.4 Eating-disorder symptoms and restrictive practises

Before recommending weight loss, fasting, carbohydrate restriction or other restrictive dietary changes, nurses should consider current or previous eating-disorder symptoms and behaviours. Relevant areas include anorexia nervosa, bulimia nervosa, binge-eating disorder, recurrent loss-of-control eating, self-induced vomiting, laxative or diuretic misuse, compulsive exercise, marked dietary restriction and severe concerns about body weight or shape. Brief screening tools such as SCOFF or BEDS-7 may identify patients who require further assessment but do not establish a diagnosis (31, 32). Purging, rapid weight loss, syncope, electrolyte abnormalities, marked restriction, recurrent binge–purge behaviour or suspected refeeding risk should prompt specialist assessment (11). A history of disordered eating does not preclude nutritional support, but it changes its priorities. Adequacy, regularity, flexibility and reduction of harmful restrictive behaviours generally take precedence over weight-focused advice. Calorie counting, rigid food rules and frequent self-weighing should not be routine nursing interventions in patients vulnerable to restrictive or compulsive eating.

### 5.5 Medication, physical health and metabolic risk

Medication use and relevant physical comorbidities should be included in nutritional assessment. Antidepressants and other psychotropic medicines may affect appetite, body weight, gastrointestinal function and metabolic health, while dietary changes and supplements may influence treatment tolerability or safety (33). Nurses should document prescribed medicines, over-the-counter products and nutritional supplements and note changes in eating behaviour or physical symptoms that may be treatment-related. Relevant comorbidities include diabetes, dyslipidaemia, hypertension, cardiovascular disease, renal or hepatic disease, gastrointestinal disorders, thyroid disease and anaemia. Weight, blood pressure and available metabolic or laboratory results may be monitored according to local protocols. Decisions regarding specific investigations and diagnostic interpretation remain the responsibility of the appropriate clinician. Nonspecific symptoms such as fatigue, cognitive difficulty or low energy should not automatically be attributed to nutritional deficiency. Where deficiency is suspected, the nursing role is to identify and communicate relevant symptoms and risk factors and facilitate further assessment. Diagnosis, investigation of the cause and targeted treatment of iron, vitamin B12, folate, vitamin D or other deficiencies should follow appropriate medical or specialist evaluation. In patients with malnutrition and reduced mobility, skin integrity and pressure-injury risk also warrant attention because nutritional status may affect both pressure-injury development and wound healing (34).

### 5.6 Food access and functional capacity

Nutritional advice has limited value if it cannot be implemented in everyday life. Assessment should therefore include food affordability, access to shops or meal services, transport, food storage and cooking facilities, social support and the patient’s ability to obtain, prepare and eat food. Anergia, impaired concentration and executive dysfunction may make complex meal preparation unrealistic even when recommendations are understood. Nursing support may therefore involve simplifying previously agreed advice and identifying practical options such as readily available foods, simple meal routines or assistance from family or community services. Occupational therapy, social care or other services may be more appropriate than additional dietary education when functional or socioeconomic barriers are the main problem. Recommendations should also take account of cultural background, financial circumstances, sensory preferences, disability and living environment (35).

### 5.7 Dietary quality and achievable goals

Nurses may explore overall dietary patterns without undertaking detailed nutrient analysis. Relevant areas include the frequency and variety of fruit and vegetables, whole-grain foods, legumes, nuts and seeds, protein sources, and consumption of highly processed foods and sugar-sweetened beverages. The purpose is to identify areas that could reasonably be improved and barriers to adequate eating rather than to classify foods as inherently “good” or “bad.” When change is appropriate, a small number of jointly agreed and achievable goals is preferable to extensive dietary restructuring. Examples include establishing a more regular eating pattern, adding a protein source to a main meal, increasing access to fruit or vegetables, or gradually reducing reliance on highly processed foods where appropriate. Goals should be reviewed during follow-up and modified if they become difficult to maintain, unnecessarily restrictive or compromise adequate intake (8, 36).

### 5.8 Supplements and self-directed dietary practises

Supplement use should be routinely assessed because patients may not regard vitamins, herbal preparations, protein products or other nutraceuticals as medication. Evidence for nutraceuticals should also be distinguished from general food-based recommendations. Although WFSBP/CANMAT guidance discusses selected nutraceuticals as adjunctive treatments for depressive disorders, this does not establish efficacy in preventing recurrence after TRD (10). The nursing role is to identify and document supplement use and support its safe integration with the wider treatment plan rather than to initiate supplement regimens independently. Assessment should include the product, dose, indication, source of recommendation, concurrent use of other preparations and whether the prescribing clinician is aware of its use. Supplementation should not replace assessment of dietary adequacy or investigation of suspected deficiency. Particular caution is required with products that have relevant pharmacological effects or potential drug interactions, including St John’s wort, and with multiple supplements of uncertain composition. Suspected adverse effects, potential interactions, high-dose use, pregnancy, renal or hepatic disease or concerns about mood activation should prompt review by an appropriately qualified clinician (10, 37–42). Restrictive diets, prolonged fasting, rapid weight-loss practises and extensive supplement use should likewise trigger further assessment of nutritional risk. Detailed dosing recommendations are not provided here because these would imply a prescribing role outside routine nursing practise. Where supplementation is clinically indicated, dosing and monitoring should follow the relevant medical, pharmaceutical or dietetic protocol.

### 5.9 Ongoing monitoring and escalation of care

Nutritional care should be reviewed over time rather than delivered as a single episode of advice. Follow-up may address whether agreed actions were feasible, whether appetite or intake has changed, whether weight trajectory is concerning and whether new practical barriers or nutritional risks have emerged. When changes in eating behaviour form part of the patient’s usual pattern of deterioration, nutritional warning signs may also be included in the relapse-prevention plan. Additional clinical or specialist input is required when nutritional problems exceed the scope of routine nursing care. Examples include suspected malnutrition or eating disorder, inability to maintain adequate intake, rapid or unexplained weight change, recurrent purging, possible refeeding risk, metabolic or medical instability, persistent gastrointestinal symptoms, complex therapeutic dietary needs, potential nutrient–drug interactions or persistent nutritional difficulties despite basic support (11). Suspected refeeding risk should prompt timely medical and dietetic assessment. The nursing role is to recognise and communicate relevant risk factors, support monitoring and implement the agreed multidisciplinary care plan rather than independently determine a refeeding regimen (30). Depending on the problem, management may involve a registered dietitian, physician, psychiatrist, eating-disorder service or another member of the multidisciplinary team. Specialist involvement does not remove the nursing role. Nurses remain involved in observing change, supporting implementation of the agreed plan, identifying barriers and communicating relevant information across the care team.

## 6 General nutritional guidance within nursing care

The first priority is adequate intake rather than dietary optimisation. Weight-loss or restrictive advice should not be prioritised in patients with markedly reduced intake, unintentional weight loss or highly irregular eating. Nursing support can instead reinforce adequate food and fluid intake, regular opportunities to eat and simple measures to improve meal adequacy.

When executive functioning is impaired, accessibility and predictability may matter more than dietary complexity. Practical options may include simple or ready-to-eat foods requiring little preparation and acceptable to the patient. Advice should also account for dental, swallowing, gastrointestinal, sensory and cultural needs.

Markedly reduced intake or substantial recent weight loss should prompt consideration of refeeding risk and appropriate medical or dietetic assessment. Medical assessment and multidisciplinary management take priority when there is marked weakness, syncope, dehydration, hypotension, bradycardia, oedema, electrolyte disturbance or inability to maintain adequate oral intake.

Once adequate intake has been established, general healthy-diet principles can be introduced. The depression literature provides the most consistent support for Mediterranean-style dietary patterns, although most studies have examined depressive symptoms rather than recurrence (14–19, 33, 43). Practical advice may include greater intake of vegetables and fruit, legumes, whole grains, nuts and seeds, unsaturated fats and adequate protein, together with reduced frequent consumption of sugar-sweetened beverages and energy-dense, nutrient-poor foods. The overall pattern may be plant-forward without requiring an exclusively plant-based diet and should be adapted to the patient’s preferences, cultural background, medical needs and practical circumstances (8).

Dietary changes should be introduced at a pace the patient can tolerate. Large increases in fibre-rich foods, for example, may need to be gradual in patients with low habitual fibre intake or gastrointestinal symptoms. Hydration and gastrointestinal tolerance should be reviewed where relevant.

Ultra-processed foods are better discussed in terms of practical substitutions than prohibition. Observational studies have associated higher consumption of ultra-processed foods with adverse depression-related outcomes, but these data do not establish a causal relationship with relapse (36). Processing alone is also an imperfect marker of nutritional quality: frozen vegetables, canned legumes, whole-grain bread and fortified products may be practical and nutritionally useful. Moralising labels such as “clean,” “good” or “bad” foods should be avoided.

Hydration, caffeine and alcohol should be considered when they affect sleep, anxiety, appetite, treatment adherence or safety. Advice on caffeine should take habitual intake and potential withdrawal effects into account rather than routinely recommending abrupt cessation. Alcohol and other substance use should be addressed as part of the broader psychiatric assessment, particularly when they interfere with eating, treatment, sleep or daily functioning.

In patients with cardiometabolic disease, emphasis can be placed on carbohydrate quality rather than routine carbohydrate elimination. General advice may favour whole grains, legumes, vegetables and other minimally processed carbohydrate sources over refined carbohydrates and sugar-sweetened beverages. More substantial carbohydrate restriction, particularly in patients receiving glucose-lowering medication, requires medical and dietetic oversight because it may alter glycaemic control and medication requirements. Very-low-carbohydrate and ketogenic diets should not be included in routine nursing nutritional guidance for TRD. Emerging studies, including a recent randomised trial in TRD, warrant further investigation but do not establish ketogenic dietary treatment as standard continuation or maintenance care (44–49). Current findings should therefore be considered preliminary. If a ketogenic intervention is being considered, specialist assessment, medication review, eating-disorder safeguards and dietetic supervision are required, together with appropriate monitoring of nutritional adequacy, metabolic and gastrointestinal effects, hydration and other potential adverse outcomes.

## 7 Discussion

Nutritional care can form part of psychiatric nursing practise in TRD, although evidence specifically evaluating nurse-delivered nutritional interventions in this population is scarce. The limited number of trials should not be taken to mean that nutritional assessment and monitoring fall outside mental health nursing. These activities overlap with established nursing responsibilities in health promotion, self-care support, treatment monitoring, patient education, care coordination and interdisciplinary practise (50). Studies from broader psychiatric populations also show that mental health nurses can deliver structured behavioural and lifestyle interventions, although nutrition-specific evidence remains limited (25).

This is relevant to TRD because nutritional problems frequently coexist with residual depressive symptoms, impaired functioning, prolonged pharmacological treatment and physical comorbidity. Changes in appetite, meal regularity, body weight or ability to obtain and prepare food may reflect medication effects, residual symptoms, poorer self-care or emerging physical illness. Nutritional assessment and longitudinal monitoring can therefore complement routine psychiatric follow-up without constituting specialist nutritional treatment.

The limits of this role need to remain explicit. Psychiatric nurses can screen for nutritional risk, identify changes in eating behaviour and physical health, provide general dietary education, reinforce recommendations agreed with the multidisciplinary team, support self-management and coordinate referral. Individualised therapeutic diets, diagnosis and treatment of malnutrition or eating disorders, refeeding management and complex nutrient–drug interactions require specialist dietetic, medical or pharmaceutical expertise. The purpose of nursing involvement is not to transfer specialist nutritional treatment to nursing staff, but to improve recognition, implementation and continuity of care.

The exact boundaries of nutritional care within psychiatric nursing will differ between healthcare systems. Scope of practise, level of autonomy and referral requirements are shaped by local regulation, professional standards and service organisation. The activities described here should therefore be adapted to the responsibilities assigned to psychiatric nurses in a given setting. This is particularly important for tasks that may sit close to the boundary between routine nursing assessment and specialist nutritional or medical care. A further consideration is whether nurses have sufficient preparation to take on these responsibilities in practise. Limited knowledge of nutritional medicine has been reported amongst other mental health professionals (13), while the extent to which nutrition is incorporated into psychiatric nursing education is less clear. If nutritional screening and basic dietary support are to become a more consistent part of TRD follow-up, nurses may need additional training in areas such as nutritional risk, eating-disorder warning signs, refeeding risk, supplement use and indications for referral. This may be addressed through continuing professional education and, in the longer term, through greater inclusion of nutrition-related competencies in psychiatric nursing curricula. The feasibility of these activities also depends on staffing, caseloads and service organisation. Brief nutritional screening can be incorporated into routine nursing assessment, with more detailed monitoring reserved for patients with identified risks. Clear referral pathways are important to avoid adding workload without appropriate follow-up.

The way nutritional issues are discussed is also important from the patient perspective. During depressive episodes, reduced motivation, low self-confidence and difficulties with planning or maintaining daily routines can make changes in eating habits difficult to introduce and sustain. Advice should therefore be realistic and adapted to the patient’s current functioning, rather than adding another source of pressure. In broader psychiatric populations, weight-focused discussions may also be experienced as uncomfortable or stigmatising. For this reason, conversations about nutrition should not concentrate on body weight alone, but also include eating patterns, practical barriers, physical health and the patient’s own priorities (51, 52).

Continuity is particularly relevant in TRD, where patients often require prolonged follow-up and may experience fluctuating residual symptoms or repeated deterioration. Regular nursing contact may allow earlier recognition of progressive appetite loss, increasing meal omission, rapid weight change, recurrent restriction or bingeing, problems obtaining or preparing food, or declining ability to maintain established routines. Therapeutic engagement, patient education and empowerment are established components of psychiatric nursing (53) and the therapeutic relationship may make it easier to discuss eating behaviour and other aspects of self-care that are closely linked to motivation and functioning (24).

Clear escalation pathways are essential. Rapid weight loss, prolonged minimal intake, recurrent purging, electrolyte abnormalities, suspected eating-disorder pathology, metabolic instability or possible refeeding risk require specialist assessment. When refeeding risk is suspected, nurses should recognise and communicate relevant risk factors, support monitoring and implement the agreed multidisciplinary plan rather than determine the refeeding regimen independently. ASPEN identifies low BMI, substantial recent weight loss, markedly reduced intake, loss of muscle or fat mass and abnormalities in phosphate, potassium or magnesium amongst relevant risk factors (30).

Nutritional care in TRD should be integrated with continuation and relapse-prevention treatment rather than presented as an independent means of preventing recurrence. Its potential value lies in supporting physical health, daily structure, self-management and treatment adherence. When appetite, meal regularity or food-related self-care form part of an individual patient’s pattern of deterioration, these changes may also be useful warning signs within the relapse-prevention plan.

**Figure 1:**
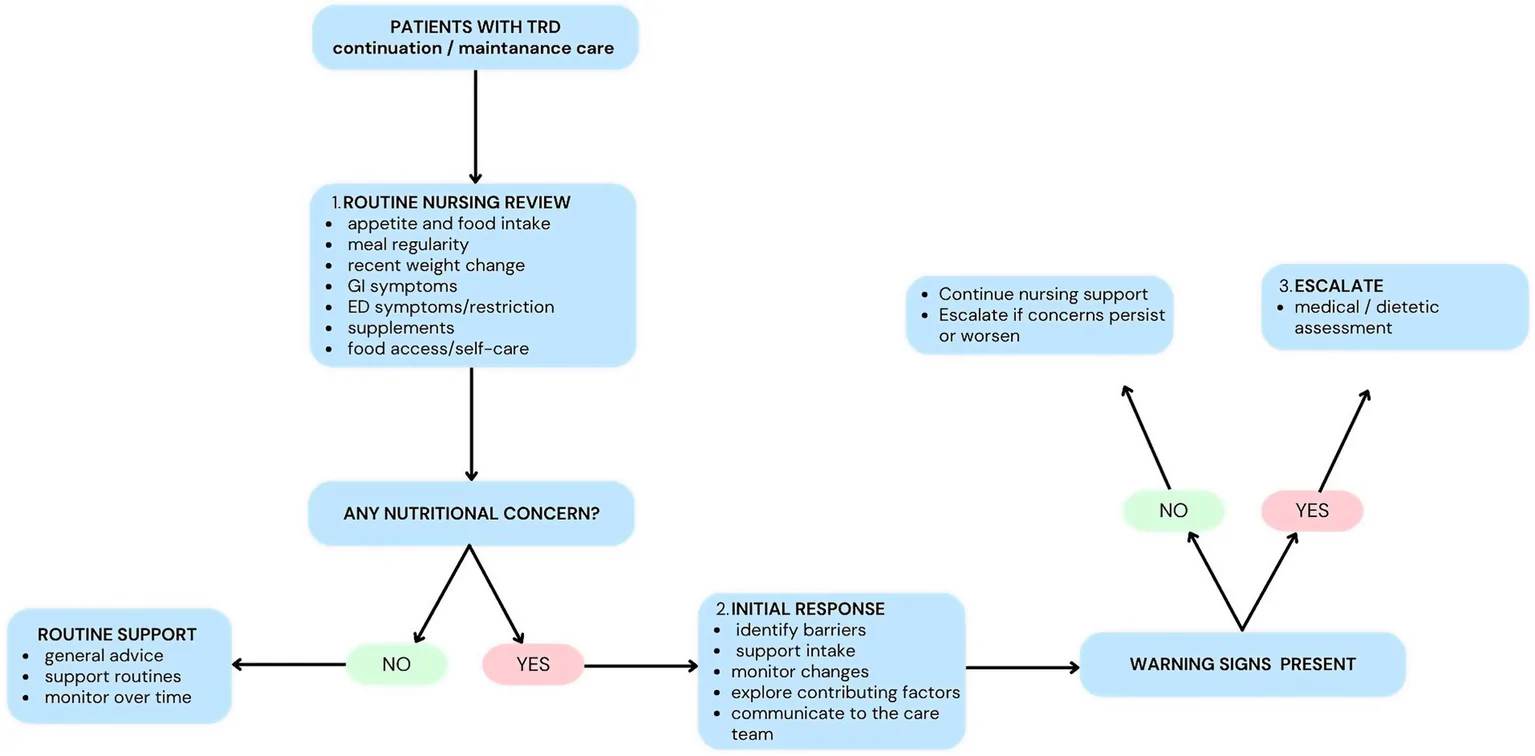
The figure summarises a practical approach to nutritional assessment, initial nursing support, monitoring and escalation in patients with TRD during continuation and maintenance care. It is intended as a quick-reference tool based on the broader guidance presented in Table 1. Escalation refers to further assessment by the appropriate healthcare professional, such as a physician, dietitian, or other specialist, depending on the identified problem.

Follow-up should be adjusted to clinical need, with closer review after hospital discharge, major medication changes, rapid weight change or deterioration in intake. Relevant areas include appetite, meal regularity, gastrointestinal symptoms, sleep, energy, functional self-care, substance use and the feasibility of agreed goals. A simplified nursing pathway for routine assessment, monitoring and escalation is presented in Figure 1. Weight, metabolic parameters and laboratory findings should be monitored when clinically indicated and interpreted in the wider clinical context. When nutritional warning signs emerge, nursing care should focus on identifying barriers, assessing whether they accompany broader psychiatric deterioration, reinforcing previously agreed strategies and involving other members of the multidisciplinary team when required.

Several limitations should be considered when interpreting this review. Evidence directly examining the effect of nutritional interventions on relapse or recurrence in TRD remains very limited, and the available data are largely drawn from broader depression literature. In addition, the proposed role of psychiatric nurses is partly informed by studies conducted in populations with schizophrenia or other serious mental illnesses rather than TRD specifically. Differences in symptom profiles, pharmacological treatment, functional impairment and care settings may limit the extent to which these findings can be transferred directly to patients with TRD. The recommendations presented here should therefore be viewed as clinically informed guidance based on the best available adjacent evidence rather than as interventions specifically validated for relapse prevention in TRD.

## 8 Conclusion

Nutritional care can be incorporated into continuation and maintenance treatment for TRD as an adjunct to relapse-prevention and recovery-oriented care. Current evidence does not support diet as a stand-alone strategy for preventing depressive recurrence, but nutritional assessment and follow-up may help address modifiable problems related to physical health, residual symptoms, self-care and treatment adherence. Psychiatric nurses can contribute through nutritional risk screening, general dietary education, longitudinal monitoring and coordination of specialist care. The scope of these activities should reflect local professional regulations, available competencies and service resources. Clear professional boundaries are essential: malnutrition, eating disorders, refeeding management, therapeutic diets and complex nutrient–drug interactions require appropriately qualified dietetic or medical input. In practise, brief nutritional screening may be incorporated into routine nursing care, with more detailed assessment reserved for patients with identified risks. A structured multidisciplinary approach, supported by clear referral pathways, may allow nutritional care to be integrated safely and realistically into routine TRD management while preserving continuity of care.
